# Thermo-Reactively Coupled (TRC) HDPE for Melt-Processable Warp-Resistant FDM 3D Printing

**DOI:** 10.3390/polym18172169

**Published:** 2026-09-05

**Authors:** Subhaprad Ash, Muhammad Naveed, Muhammad Rabnawaz

**Affiliations:** School of Packaging, Michigan State University, 448 Wilson Road, East Lansing, MI 48824, USA; ashsubha@msu.edu (S.A.); naveedmu@msu.edu (M.N.)

**Keywords:** polyethylene, thermo-reactively coupled (TRC), fused deposition modeling (FDM), reactive extrusion, ester-linked interchains, 3D printing, melt-processable, additive manufacturing, filament formation

## Abstract

Polyethylene, the most widely used and inexpensive commodity thermoplastic, remains unsuitable for 3D printing via fused deposition modeling (FDM) because of shrinkage-induced warping. Herein, this study aimed to develop a single-step reactive extrusion process to improve the filament-forming ability and 3D printability of high-density polyethylene (HDPE). HDPE was reacted with maleic anhydride, 1,10-decanediol, and a zinc acetate catalyst via peroxide-driven grafting in a melt extruder, and the subsequent coupling reactions yielded ester-containing, modified polyethylene structures. Unlike unmodified HDPE, which produced irregular filaments and poor 3D printing, the modified HDPE formulations produced filaments with a consistent diameter and enabled successful FDM printing with improved print quality. The optimized carbon-black-containing modified HDPE systems exhibited substantial mechanical improvements, with average enhancements of approximately 50–75% in tensile stress at break and an approximately 200% increase in elongation at break compared with neat HDPE. These results demonstrate that ester-containing HDPE has the potential to convert commodity HDPE into a melt-processable and mechanically improved feedstock for additive manufacturing applications. This study suggests that widely available post-consumer pigmented HDPE, which is otherwise unsuitable for reuse in packaging, could potentially be explored for 3D printing as an alternative to expensive acrylonitrile butadiene styrene (ABS) and polylactic acid (PLA).

## 1. Introduction

The objective of this work is to develop a scalable reactive extrusion process that converts commodity high-density polyethylene (HDPE) into a filament having a consistent diameter and improved tensile performance for fused deposition modeling (FDM). HDPE remains challenging to process reliably by FDM, leaving a vast, underutilized waste resource that is landfilled today. HDPE is a go-to material for commodity and industrial applications, spanning from packaging containers and pipes to automotive components and construction materials [1]. Global polyethylene (PE) production has surpassed 116 million metric tons annually as of 2025, and is projected to exceed this, with expectations to reach a total market volume approaching ~178 million metric tons by 2035 [2,3]. More than one-third of all global plastic is PE [1,4], with HDPE dominating the PE production by accounting for over 42% of the total, while low-density polyethylene (LDPE) along with linear low-density polyethylene (LLDPE) make up the remainder [3]. Moreover, post-consumer HDPE waste presents an enormous underutilized resource that aligns with the growing demand for circular economy strategies in polymer processing.

Additive manufacturing, particularly extrusion-based techniques such as fused filament fabrication (FFF), which is also referred to as fused deposition modeling (FDM), has transformed the way polymeric products are prototyped and manufactured [5,6,7]. FDM enables customized parts to be produced layer by layer without requiring molds or machining, thus making it an attractive platform for industries ranging from aerospace to biomedical devices [8]. FDM is the most widely adopted additive manufacturing technology for thermoplastics due to its low equipment cost, material versatility, and ease of operation [9]. Despite this, the portfolio of polymers routinely used in FDM is still dominated by a relatively narrow set of conventional thermoplastics, examples of which include polylactic acid (PLA) and acrylonitrile butadiene styrene (ABS). HDPE, which constitutes the largest fraction of the global plastics market, is unsuitable for 3D filament formation [10]. Three primary barriers limit the printability of HDPE. First, warpage and dimensional instability arise from HDPE’s high crystallinity (60–80%) and the associated large volumetric shrinkage (~5–6%), most of which occurs near 115–120 °C—that is, after each deposited road has already fused to the underlying layer. The constrained contraction generates through-thickness residual stresses that curl the printed part and lift it from the build plate [11,12]. Second, poor interlayer adhesion results from rapid crystallization at the road interface, which solidifies the deposited strand before sufficient chain interdiffusion and entanglement can develop across the weld. The low surface energy and non-polar nature of HDPE further weaken interfacial bonding. Third, a narrow and challenging melt-processing window results from HDPE’s low melt strength and absence of strain hardening, arising from its linear, unbranched architecture. The melt cannot sustain drawdown without necking or draw resonance, yielding filaments of inconsistent diameters [13]. Consequently, these dimensional stability issues render the production of accurate, warp-free parts extremely difficult without extensive parameter optimization or the use of heated enclosures. However, developing facile, solvent-free, scalable, and commercially viable strategies that employ 3D printing to process PE waste can offer an environmentally sustainable solutions enabling the production of sustainable components, while also delivering cost savings.

Blending recycled HDPE with linear low-density polyethylene (LLDPE) has been reported as a facile approach to reduce overall crystallinity and increase toughness [14]. Recycled HDPE (rHDPE)/LLDPE blends showed a reduction in warpage, but it also trades off the strength and modulus of the HDPE. Researchers have investigated carbon-based nanoparticles (examples of which include graphene nanoplatelets (GNPs) and carbon nanotubes (CNTs)) to improve both polymer processability [11,15,16,17] and the physical properties of polymers [18,19,20,21,22]. Despite these efforts, blending and nanofiller approaches are physical modifications of an intrinsically thermoplastic, topologically unconstrained matrix. They can dilute or mechanically restrain crystallization shrinkage, but they cannot relax the residual stress once generated, cannot promote covalent bonding across the deposited road interface, and—in the case of nucleating fillers—may even accelerate the crystallization they are intended to counteract. Moreover, practical challenges, including nanoparticle agglomeration, low compatibility with non-polar HDPE, and the high cost and specialized processing required for uniform dispersion, further limit their scalability [23,24,25,26]. This intrinsic limitation of physical fillers motivates the exploration of a material-level reactive modification strategy that may provide a more effective route for improving the printability and mechanical performance of HDPE.

A more recent approach involved incorporating dynamic covalent bonds into the polyethylene backbone to create vitrimeric or thermally reversible networks [27,28,29]. Unlike conventional crosslinked networks, which are permanently set once formed and cannot be reprocessed, or dissociative dynamic networks (e.g., Diels–Alder adducts) that sharply de-crosslink into small molecules, vitrimers are permanent networks in which covalent bonds undergo exchange without a net loss of crosslink density [30,31,32]. This exchange proceeds through associative mechanisms such as transesterification, disulfide metathesis, silyl ether, vinylogous urethane exchange, or dynamic phenolic urethane linkages and is typically accelerated by a catalyst or elevated temperature. Montoya-Ospina et al. reported the screw-assisted 3D printing of HDPE vitrimers (HDPE-V) and demonstrated that the dynamic crosslinks reduced shrinkage during printing and, upon post-print annealing, decreased mechanical anisotropy relative to neat HDPE [33]. In a separate study, Harmon et al. investigated HDPE vitrimers prepared by reactive blending of maleic anhydride-grafted polyethylene with dithiodianiline [34]. The resulting vitrimer could be printed with reduced warpage while maintaining melt and crystallization temperatures similar to those of the parent HDPE. In parallel, thermally reversible crosslinked polyethylene networks based on furan–maleimide Diels–Alder (DA) chemistry have been constructed via solvent-free melt processing. These networks exhibit thermal switching of the network structure: the DA adducts dissociate at elevated temperature (retro-DA), enabling reprocessing, and reform upon cooling, restoring the crosslinked state [32,35]. Despite these advancements, existing approaches often require furan-functionalization or specialty-synthesized disulfide/dithioaniline crosslinkers, raising occupational safety and end-use concerns due to the potential risk of toxic release during high-temperature extrusion and printing. Therefore, there remains a clear and unmet need for a facile modification of polyethylene that is constructed from readily available materials, proceeding via a simple solvent-free melt process, to make polyethylene 3D printable with improved printing quality. Such an approach would avoid hazardous reagents, minimize synthetic complexity, and operate entirely in a solvent-free melt process, thereby offering a safer, more scalable, and economically attractive pathway for transforming waste polyethylene into a high-performance 3D-printing feedstock. For example, maleic anhydride is a commodity chemical that is already produced at industrial scale, and its grafting onto polyethylene via peroxide-initiated reactive extrusion is a mature, well-understood process that can be directly integrated into existing compounding infrastructure [36,37,38]. Combining this established grafting platform with a similarly low-cost, non-hazardous coupling agent—such as an aliphatic diol—would therefore constitute a practical, scalable, and economically viable route to modify polyethylene for 3D printing applications without the need for specialty monomers or additional multi-step functionalization steps.

Improved filament diameter consistency during the extrusion-based filament-making process is a critical prerequisite for reliable FDM printing. Although previous studies have individually addressed the challenges through build-plate adhesion, polymer blending, nanofiller reinforcement, or dynamic polymer networks, a simple reactive-extrusion strategy capable of simultaneously achieving improved printing quality and mechanical properties in a single HDPE-based material has remained largely unexplored. To address this knowledge gap, we report a novel approach to prepare modified HDPE for FDM 3D printing through a single-step reactive extrusion of HDPE with a pre-designed additive containing mainly maleic anhydride, 1,10-decanediol, and zinc acetate as a catalyst, along with other co-ingredients, enabling in situ grafting and coupling reactions that produce ester-containing interchain-coupled polyethylene structures while preserving the melt-processable nature of HDPE. The specific contribution of this work is the development of a simple solvent-free modification strategy aimed at improving filament diameter consistency and FDM printability, while evaluating the influence of low carbon-black loading on the resulting mechanical response. The resulting material was evaluated in terms of filament fabrication, thermal behavior, FDM printability, and tensile performance. This work provides a scalable and economically viable pathway to potentially convert post-consumer HDPE waste into a high-performance, melt-processable 3D printing material, thereby bridging the gap between the world’s most abundant commodity plastic and the rapidly expanding additive manufacturing industry.

## 2. Materials and Methods

### 2.1. Materials

Maleic anhydride (MA, 99%), dicumyl peroxide (DCPO, 98%), 1,10-decanediol (DDO, 98%), zinc acetate (ZA, 99.9%), *N,N′*-ethylenebis(stearamide) (EBSA, amine blend beads, <840 μm), 4-hydroxy-2,2,6,6-tetramethylpiperidine 1-oxyl (4-hydroxy-TEMPO, 97%), 1,1,2,2-tetrachloroethane-d_2_ (≥99.5 atom % D), and carbon black were purchased from MilliporeSigma (St. Louis, MO, USA) and used without further purification. The organic chemicals, including MA, DCPO, and DDO were characterized by ^1^H nuclear magnetic resonance (NMR) spectroscopy prior to use. Two grades of high-density polyethylene (HDPE) were used in this study. HDPE DMDA-8904 NT 7 (PE-1; MFI~4.4 g/10 min at 190 °C/2.16 kg) and HDPE DOWLEX™ IP 10262 (PE-2; MFI~10 g/10 min at 190 °C/2.16 kg) were purchased from Dow Chemical (Midland, MI, USA).

### 2.2. Reactive Extrusion and Pelletization

HDPE was extruded with a pre-designed additive using a single-step reactive extrusion process to improve its filament-forming ability and suitability for fused deposition modeling (FDM) 3D printing. The additive was prepared as a masterbatch by thoroughly mixing the following ingredients in specific amounts: maleic anhydride (8.4 g), dicumyl peroxide (0.75 g), 1,10-decanediol (12 g), zinc acetate (2 g), *N,N′*-ethylenebis(stearamide) (2 g), and 4-hydroxy-TEMPO (1 g). The reactive extrusion process was conducted with the use of a DSM Xplore 15 cc Micro Extruder that had co-rotating conical twin-screws. First, HDPE and the pre-designed additive were premixed and fed into the extruder at a temperature of 185 °C in three increments. The rotation speed of the screws was kept at 150 rpm. After feeding all material, the mixture was compounded for 4 min. Then, the molten mixture was extruded as macro-filaments, and subsequently the filaments were pelletized for micro-filament preparation for 3D printing. A total batch size of 10 g was used for each formulation, and five independent reactive extrusion batches were prepared. The sample codes and their compositions are shown in Table 1.

### 2.3. 3D Printable Filament Preparation

A Filabot EX2 (Filabot, Barre, VT, USA) system was used as a filament extruder for preparing FDM filament from pelletized formulations for 3D printing. The extrusion conditions, including melt-processing temperature, screw speed, cooling airflow, and drive speed were optimized to obtain a filament of a consistent target diameter (i.e., ~1.70 mm) suitable for the subsequent 3D printing process. The filament diameter consistency was continuously manually monitored using a digital caliper. The extruded filaments were air-cooled, collected on a plastic reel spool, and stored for subsequent 3D printing. The optimized melt-processing conditions for FDM filament making consisted of a temperature of 185 °C, a cooling fan setting of 85%, and a drive speed adjusted to maintain a target filament diameter of 1.7 mm.

### 2.4. 3D Printing

**Figure 1:**
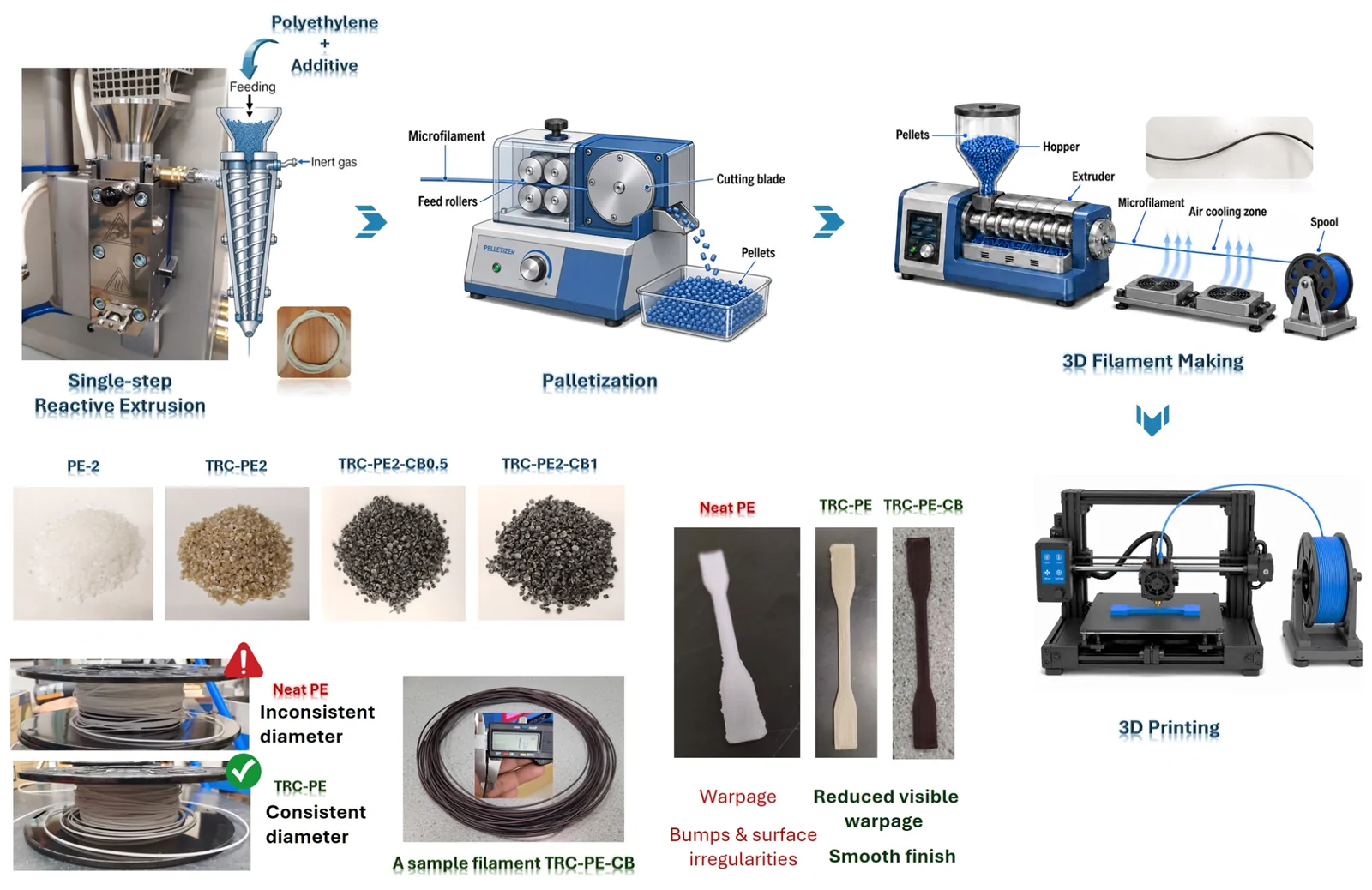
Illustration of the overall processing route in this work and representative products for the development of modified HDPE formulations (TRC-PE) for 3D printing.

3D printing of the modified HDPE was performed using a Qidi Tech X-one2 3D printer (QIDI Technology, Ruian, Zhejiang, China). The sample’s shape was designed using SOLIDWORKS^®^ software (Version 2018 SP5.0), and a corresponding 3D printable soft file for the Qidi Tech X-one2 3D printer was generated using the QidiPrint v6.5.3 Windows-based application. 3D printing was performed by employing a layer height of 0.2 mm, an infill of 50%, a print speed of 60 mm/s, a travel speed of 100 mm/s, and a minimum layer time of 30 s. The printing temperature was set at 250 °C, and the build plate temperature was heated to 120 °C throughout the printing process. A 0.4 mm MK10 brass nozzle designed for 1.75 mm filament was used for all printing experiments. All printing parameters were kept the same for all different formulations to enable a consistent comparison of their printing behavior and mechanical performance. Figure 1 depicts the graphical illustration of the overall processing route employed in this work and representative products for the development of modified HDPE formulations (TRC-PE) for 3D printing.

### 2.5. Characterization

#### 2.5.1. ^1^H NMR Analysis

A 2 g portion of the maleic anhydride-grafted HDPE (MA-*g*-HDPE) was heated in 100 mL of xylene under reflux for 4 h, prior to hot filtration into 300 mL of acetone. Subsequently, the xylene-soluble, acetone-insoluble polymer was washed with acetone before it was then dried at 60 °C under vacuum. ^1^H NMR characterization of the extracted sample was performed with a Bruker 500 MHz NMR-Spectrometer (operating frequency = 499.955 MHz) that was equipped with a 10 mm BroadBand (50–126 MHz) probe. The characterization was performed at 120 °C, and deuterated 1,1,2,2-tetrachloroethane was employed as the solvent. Prior to characterization, ~10 mg of each specimen was dissolved in 1 mL of 1,1,2,2-tetrachloroethane-*d*_2_.

#### 2.5.2. FTIR Analysis

All extruded samples were characterized by Fourier-transform infrared (FTIR) spectroscopy. A Jasco FTIR spectrometer (FTIR-6600 type, Easton, MD, USA) that had an ATR PRO ONE accessory was used to perform FTIR analysis. Spectra were recorded in the transmittance mode at room temperature over a wavenumber range of 4000–400 cm^−1^ at a resolution of 4 cm^−1^ using 32 scans per sample.

#### 2.5.3. Differential Scanning Calorimetry (DSC)

Differential scanning calorimetry (DSC) was performed for all extruded samples to investigate their thermal transitions. Under nitrogen protection, a TA Instruments DSC-Q100 (New Castle, DE, USA) thermal analyzer was used for the analysis in the heating range from 40 to 200 °C, and the temperature was elevated at 5 °C/min. The DSC curves were used to determine the melting temperature (*T*_m_), onset and endset melting temperatures (*T*_m-onset,_ *T*_m-endset_), as well as the enthalpy change of melting (Δ*H*_m_).

#### 2.5.4. Thermogravimetric Analysis (TGA)

Thermogravimetric analysis (TGA) was performed for all extruded samples to investigate their thermal stabilities and degradation behavior. A TA Instruments Q-50 thermogravimetric analyzer (New Castle, DE, USA) was used for the analysis. Approximately 6–9 mg of each sample was initially placed in a platinum sample pan and heated from 23 to 600 °C at a heating rate of 10 °C/min under a nitrogen atmosphere at a flow rate of 40 mL/min. The weight loss of the samples was recorded as a function of temperature. The onset and endset degradation temperatures (*T*_onset_, *T*_endset_), temperature at maximum degradation rate (*T*_max_), maximum rate of degradation (*R*_max_), and slopes were determined from the TGA and derivative thermogravimetric (DTG) curves using TA Universal Analysis 2000 software (version 4.5A).

#### 2.5.5. Tensile Properties

A Universal Testing Machine Instron 5565 system (Instron Corp., Norwood, MA, USA) was employed to measure the tensile characteristics of 3D-printed specimens, which were evaluated with a computer-controlled Universal Testing Machine Instron 5565 system (Instron Corp., USA) in compliance with the ASTM D-638 (Type IV) standard. The samples were tested in triplicate at a crosshead speed of 10 mm/min with a 5 kN load cell and reported values represent mean ± standard deviation. The Young’s modulus, tensile strength, and elongation at break were evaluated based on the stress–strain curves. The experimental data were acquired and analyzed using Bluehill Universal software (Version 4.25, Instron Corp.). Prior to testing, the 3D-printed specimens were conditioned at room temperature for 48 h.

## 3. Results and Discussion

**Figure 2:**
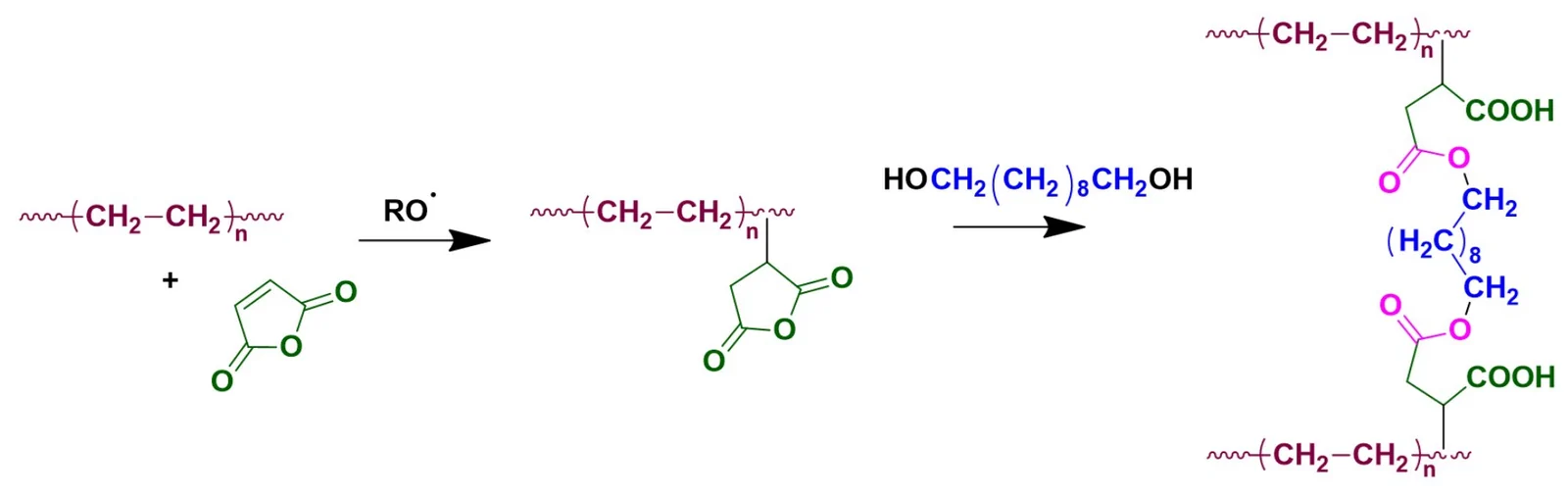
Schematic illustration depicting the proposed reactive-extrusion pathway for preparing modified HDPE. In situ maleic anhydride grafting, and subsequent coupling reactions between HDPE chains during reactive extrusion in the presence of the designed additive system.

The single-step reactive extrusion process was designed to simultaneously graft maleic anhydride onto the HDPE backbone and couple the chains through esterification with 1,10-decanediol (Figure 2). The peroxide initiates free-radical grafting of maleic anhydride, generating pendant succinic anhydride groups along the polyethylene chain. The difunctional diol then reacts with these anhydride/acid groups to form ester linkages that bridge two polymer chains, resulting in an ester-linked interchain structure. The reaction is therefore expected to produce predominantly linear ester bridges between adjacent polyethylene chains. Because the total additive loading is low (5 wt%) and maleic anhydride constitutes only a fraction of that, the number of such bridges is expected to be small. We therefore refer to the product as “modified HDPE” (TRC-PE) and describe it as containing “ester-linked interchain structures”.

### 3.1. ^1^H NMR Analysis

**Figure 3:**
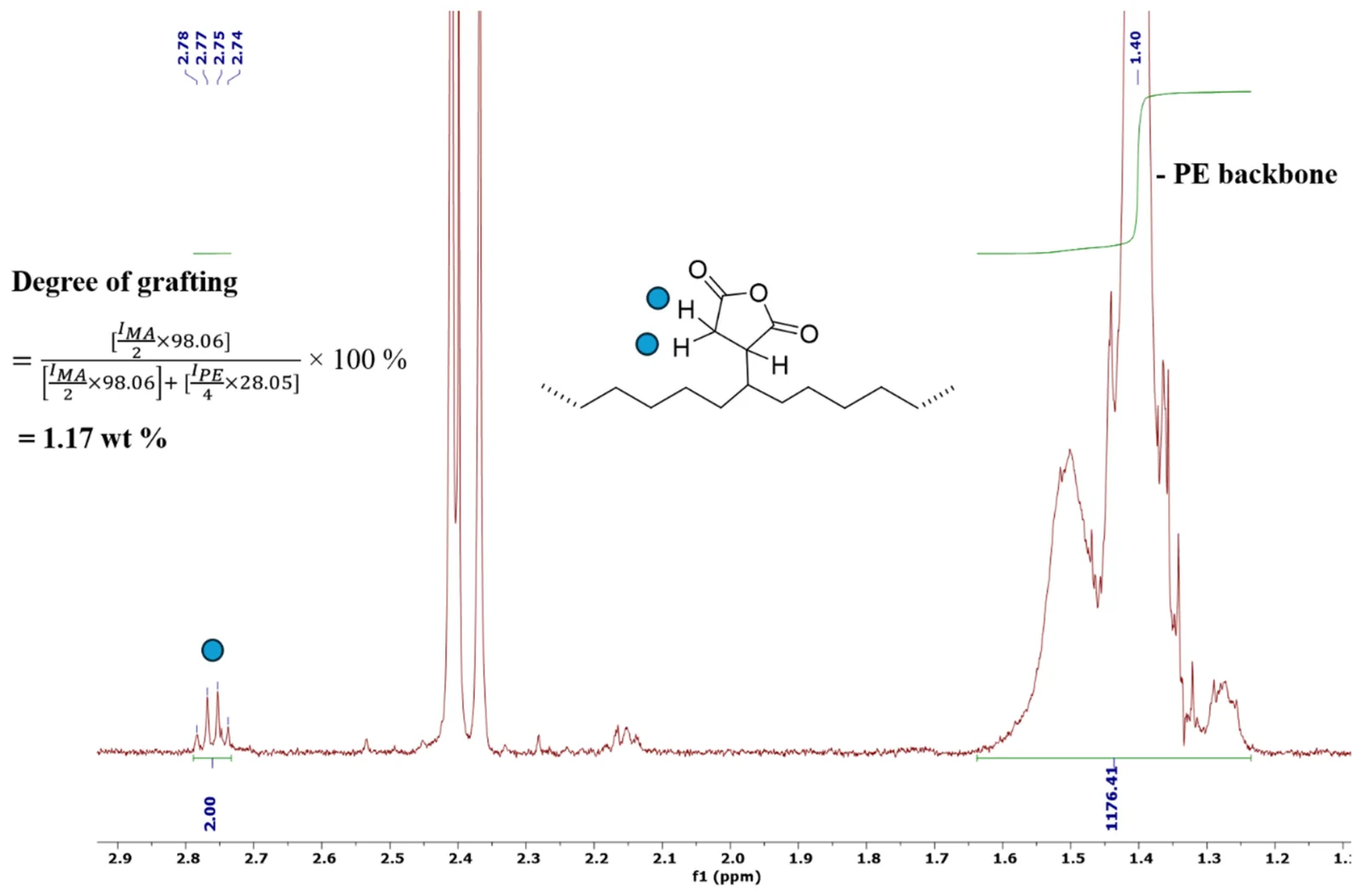
^1^H NMR spectrum of maleic anhydride-grafted HDPE (MA-*g*-HDPE).

To verify that maleic anhydride had been grafted onto HDPE, HDPE was first maleated through reactive extrusion using 1.5 wt% maleic anhydride in the presence of dicumyl peroxide (DCPO) as a free-radical initiator. The resulting maleic anhydride-grafted HDPE (MA-*g*-HDPE) was subsequently purified prior to NMR analysis to remove unreacted maleic anhydride and other low-molecular-weight species. Briefly, 2 g of the grafted HDPE was heated in 100 mL of xylene under reflux for 4 h, prior to hot filtration into 300 mL of acetone. The xylene-soluble, acetone-insoluble polymer was washed with acetone and dried under vacuum at 60 °C. The purified sample was then analyzed by ^1^H NMR spectroscopy at 120 °C with 1,1,2,2-tetrachloroethane-*d*_2_ employed as the solvent, as shown in Figure 3. The intense resonance centered at ~1.4 ppm corresponds to the methylene protons of the polyethylene backbone. The resonance observed at approximately 2.7–2.8 ppm is attributable to methylene proton environments associated with succinic anhydride structures generated by the grafting of maleic anhydride onto the HDPE chains, consistent with previous reports for maleic anhydride-grafted polyethylene [39,40,41]. The presence of these characteristic resonances after purification confirms the successful incorporation of maleic anhydride functionality onto the polyethylene backbone. The grafting density, estimated from the integration of succinic anhydride methylene resonance relative to the polyethylene backbone protons, was found to be 1.17%, as shown in Figure 3. The calculated grafting density shows good agreement relative to the initial monomer feed and corresponds to a grafting efficiency of approximately 78%, indicating that most of the monomer was successfully grafted despite some losses arising from partial monomer volatilization and competing macroradical recombination side reactions at high processing temperatures. These results demonstrate that reactive extrusion in the presence of dicumyl peroxide effectively grafted maleic anhydride onto HDPE, thereby introducing reactive functional groups necessary for the subsequent interchain coupling reactions.

### 3.2. FTIR Analysis

**Figure 4:**
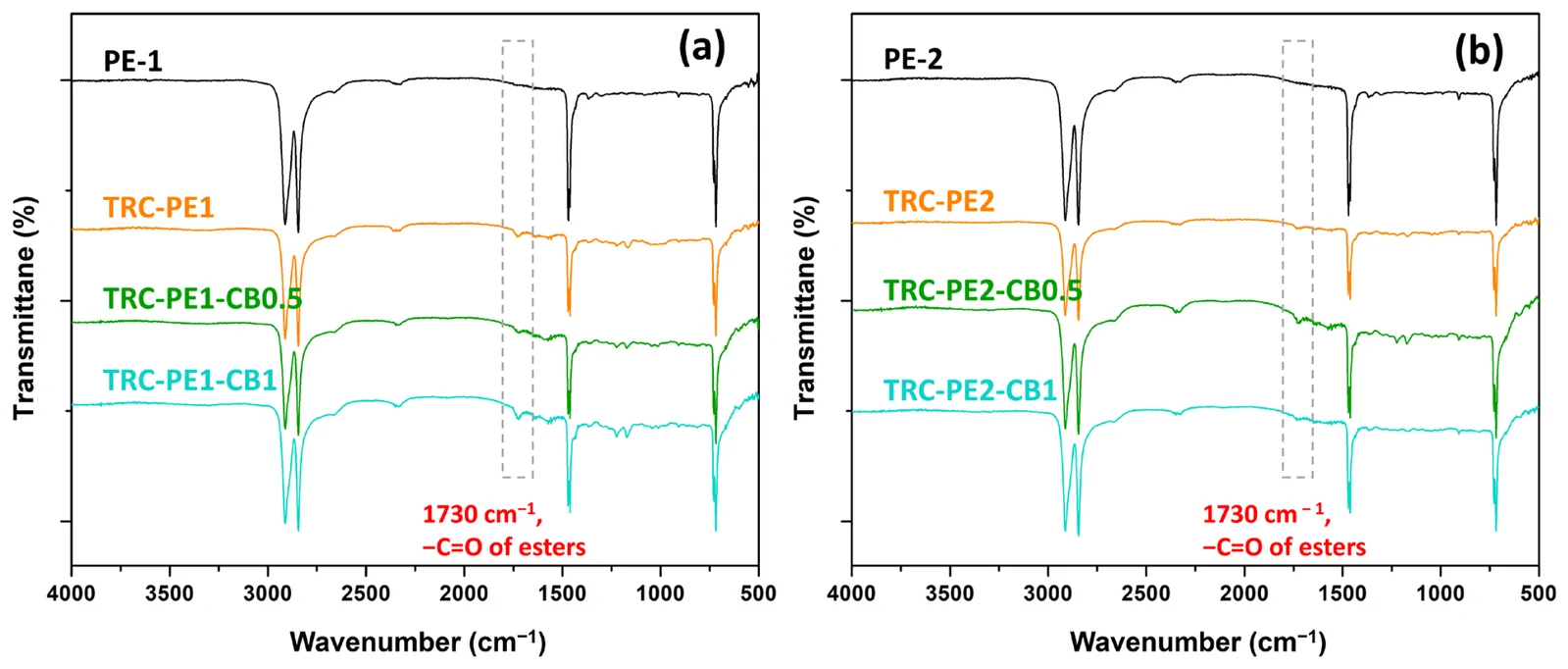
FTIR spectra of (**a**) PE-1 and TRC-PE1 formulations, and (**b**) PE-2 and TRC-PE2 formulations.

FTIR analysis of neat PE and TRC-PE formulations is presented in Figure 4. All samples exhibited the identical characteristic polyethylene absorption bands at approximately 2915 and 2848 cm^−1^ that respectively corresponded to asymmetric and symmetric CH_2_ stretching vibrations, together with the CH_2_ bending vibration near 1460–1470 cm^−1^ and the CH_2_ rocking vibration around 721–729 cm^−1^. Moreover, the peaks at 730 and 720 cm^−1^ also show −CH_2_—rocking for the polyethylene band. Following reactive extrusion, a new peak that appeared in the carbonyl region around 1730 cm^−1^ depict the C=O stretching vibrations of ester linkages, indicating the successful incorporation of oxygen-containing functionalities derived from the maleic anhydride coupling chemistry. Simultaneously, another useful change is the appearance or strengthening of bands around ~1165–1173 cm^−1^. This region is consistent with C–O stretching of ester linkages and complementarily reveals that the anhydride functionality has reacted with the hydroxyl groups of the diol to form ester-type interchain linkages. Maleic anhydride-grafted polymers often show anhydride carbonyl absorptions between 1780 and 1860 cm^−1^ [41], which is absent in our TRC systems as can be seen in the FTIR spectra of both PE1 and PE2 systems in Figure 4, and no broad OH stretching band (for 1,10-decanediol) appears in any sample, further qualitatively supporting the consumption of anhydride groups reacted with diol coupling agent during the proposed coupling reaction. The persistence of these characteristic bands in all TRC-PE formulations supports the formation of coupled structures between polyethylene chains. No substantial shifts in peak positions were observed upon carbon black addition, suggesting that carbon black primarily acts as a physical reinforcement rather than participating in additional chemical reactions. The similarity of the FTIR spectra of the PE-1 and PE-2 series indicates that both polyethylene grades underwent comparable chemical modification during reactive extrusion.

### 3.3. Differential Scanning Calorimetry (DSC)

**Figure 5:**
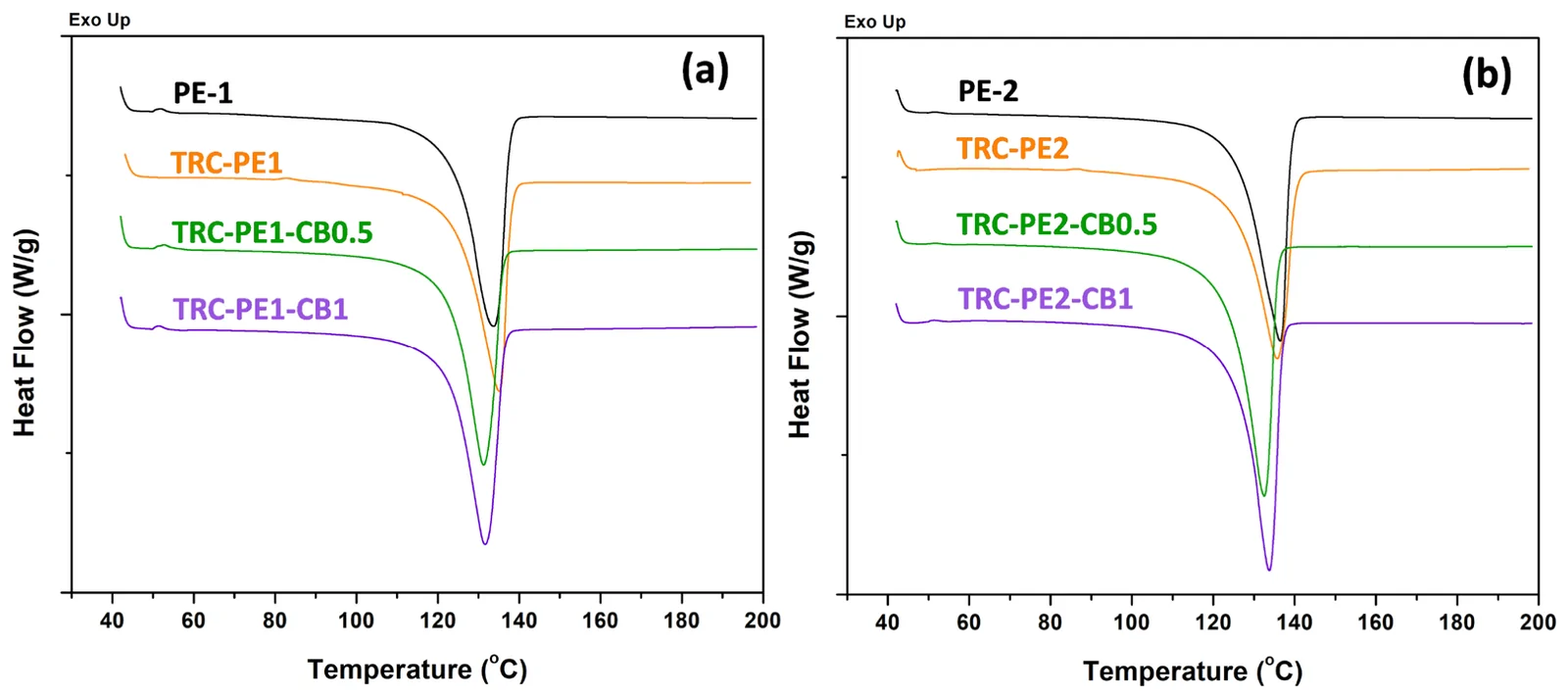
DSC curves for (**a**) PE-1 and TRC-PE1 formulations, and (**b**) PE-2 and TRC-PE2 formulations.

DSC characterization was conducted to evaluate the melting behavior of extruded TRC-PE formulations shown in Figure 5 and tabulated in Table 2. The DSC thermograms of neat HDPE and TRC-PE formulations revealed well-defined melting transitions characteristic of polyethylene. The melting temperature (*T*_m_) of neat PE-1 was approximately 133.7 °C, while TRC-PE1 exhibited a slightly higher melting temperature of approximately 135.1 °C. This slight increase in melting temperature following reactive modification suggests that the coupled structure does not adversely affect crystalline stability and may promote a more thermally stable crystalline phase. This is important because the retention of a clear melting transition supports the melt-processable nature of the modified HDPE system despite the introduction of interchain coupling structures. Furthermore, TRC-PE showed a single, symmetrical melting endotherm without an obvious secondary melting peak or high-temperature shoulder. This indicates that no clearly separated crystalline phase was formed after reactive modification, nor the bimodal distribution of crystal sizes. The carbon-black-containing formulations exhibit a modest reduction in *T*_m_ to approximately 131 °C. This may reflect a slight disruption of the crystal lattice due to the introduction of defects and reduced lamellar thickness caused by the carbon black incorporation. Importantly, these systems still maintained clear HDPE melting transitions, thus showing that the material remained thermally processable and that carbon black did not disrupt polyethylene crystallization. The melting enthalpy Δ*H*_m_ (J/g of sample mass) of TRC-PE1 formulations were observed in the order PE-1 (neat) > TRC-PE1 ≈ TRC-PE1-CB0.5, while the higher CB loading in TRC-PE1-CB1 raised the Δ*H*_m_ and partially compensated for the reduction, which suggests that CB may have imparted the heterogeneous nucleation. Furthermore, the diol and maleic anhydride grafting brought polar groups to the polyethylene chains and the introduced ester coupling structures acted as covalent tie points that partially restricted polyethylene chain packing, which may contribute to reduced visible shrinkage and warping during 3D printing. Although the degree of crystallinity was not quantitatively determined in the present study, the melting enthalpy is associated with the crystalline phase of polyethylene, and the persistence of a well-defined melting transition with minimal changes in *T*_m_ across all formulations indicates that the crystalline polyethylene phase was substantially retained. However, the crystalline phase was not eliminated, which is beneficial for preserving the thermoplastic character of HDPE. TRC-PE2 formulations exhibited similar melting characteristics and trends as those of TRC-PE1. The TRC modification in PE2 again showed a reduced Δ*H*_m_ similar to that observed in TRC-PE1. However, the TRC-PE2-CB formulations showed a prominent increase in Δ*H*_m_ after CB addition compared to neat PE2.

The most important observation is that all TRC formulations retain a well-defined melting peak within the typical HDPE melting range (~131–135 °C). This demonstrates that the fundamental melting mechanism of TRC-PE systems remains largely unchanged and the reactive modification, interchain coupling, and carbon black incorporation did not destroy the crystalline polyethylene phase. Therefore, the DSC results support the central concept that TRC-PE retains thermoplastic melt-processability while exhibiting structural modifications and the introduction of interchain coupling structures that may contribute to reduced shrinkage and improved dimensional stability during 3D printing.

### 3.4. Thermogravimetric Analysis (TGA)

**Figure 6:**
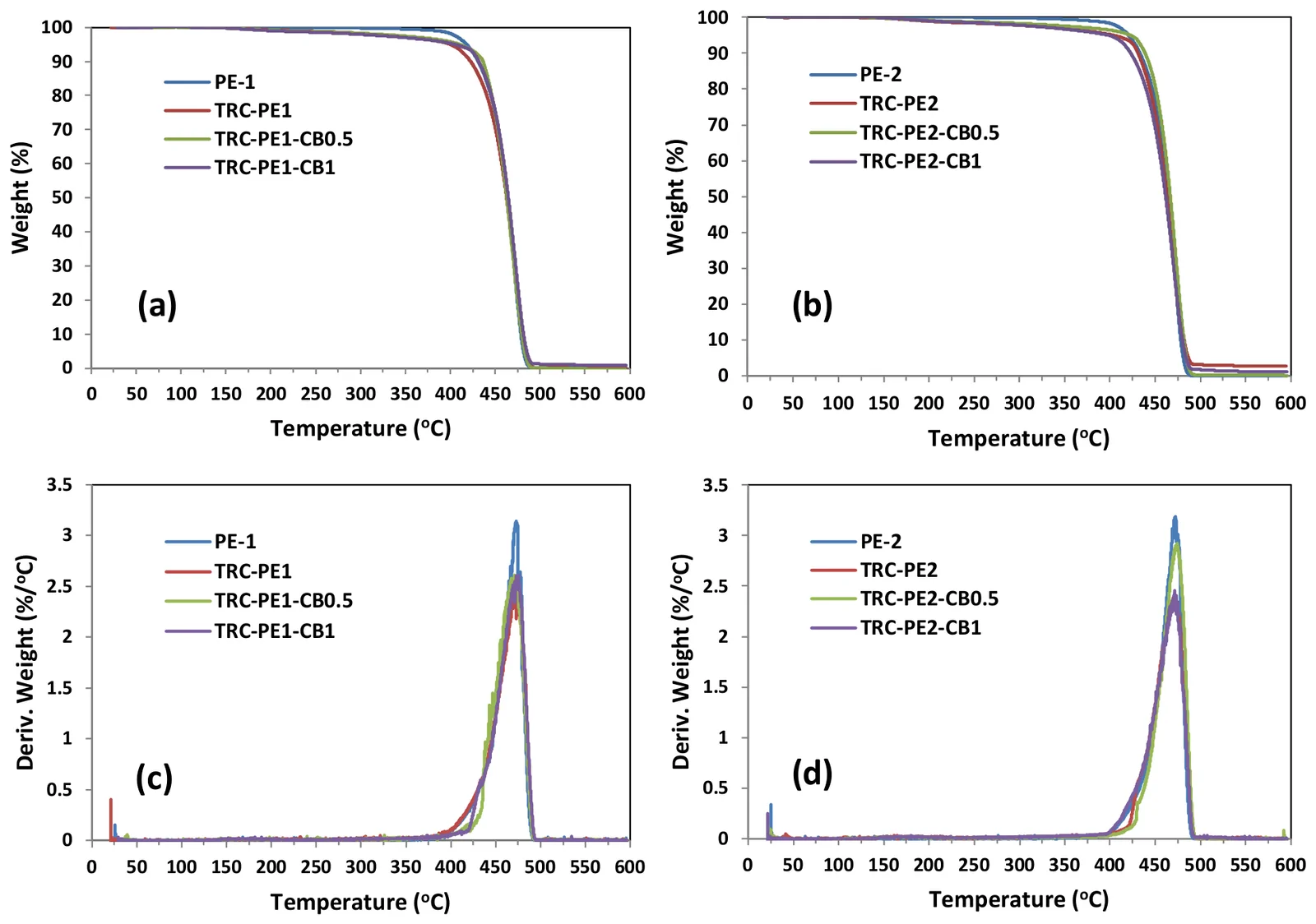
TGA curves for (**a**) PE-1/TRC-PE1 and (**b**) PE-2/TRC-PE2 formulations, and (**c**,**d**) corresponding DTG curves.

Thermogravimetric analysis (TGA) was performed to assess the thermal stability as well as the degradation characteristics of the PE and TRC-PE formulations. The important parameters, including the degradation temperatures along with the mass loss rates, are shown in Figure 6 and tabulated in Table 3. The most important finding is that all samples degrade far above the processing and 3D-printing temperatures. Even the lowest *T*_5%_ value is around 399–400 °C, while the extrusion, processing and printing temperatures were in the range of approximately 185–250 °C. Therefore, all formulations possess a large thermal safety margin for melt extrusion, filament fabrication, and FDM printing. The neat PE-1 and PE-2 formulations showed very similar degradation behavior. PE-1 exhibited *T*_max_ around 472 °C, while PE-2 also showed *T*_max_ around 472 °C. This confirms that both commercial HDPE grades undergo the typical single-step thermal degradation behavior expected for polyethylene under nitrogen. Their maximum degradation rate *R*_max_ values were also very similar, with *R*_max_ values of 3.13%/°C for PE-1 and 3.16%/°C for PE-2, indicating comparable degradation kinetics.

After TRC modification, the *T*_max_ values remain almost unchanged. TRC-PE1 and TRC-PE2 showed *T*_max_ values around 471 °C. This is a beneficial outcome because it indicates that reactive extrusion and interchain coupling do not substantially compromise the main thermal degradation resistance of the HDPE backbone. However, the early degradation temperatures decreased in the TRC systems. For example, *T*_5%_ decreases from 418 °C for PE-1 to 400 °C for TRC-PE1, and from 420 °C for PE-2 to 404 °C for TRC-PE2. The TGA-derived *T*_onset_ and *T*_endset_ describe the beginning and completion of the main mass-loss step, which further show that TRC modification broadened the degradation process. Neat PE-1 showed a TGA *T*_onset_ of 447 °C and *T*_endset_ of 480 °C, while neat PE-2 showed a *T*_onset_ of 448 °C and *T*_endset_ of 481 °C. After TRC modification, *T*_onset_ decreased to 438 °C for TRC-PE1 and 443 °C for TRC-PE2, but *T*_endset_ increased to 484 °C and 483 °C, respectively. Consequently, the degradation window, expressed as *T*_endset_ − *T*_onset_, increased from 33 °C for neat PE-1 to 46 °C for TRC-PE1, and from 33 °C for neat PE-2 to 40 °C for TRC-PE2. These results indicate that the TRC systems undergo degradation over a broader temperature range rather than showing a simple improvement in initial degradation temperature. In particular, the decrease in early-stage degradation of TRC formulations may arise from the presence of grafted maleic anhydride-derived structures, ester linkages, peroxide-derived residues, or low-molecular-weight species introduced during the reactive modification process. Another possibility could be that the ester linkages and any residual catalyst (zinc acetate) might catalyze early decomposition of these groups, but since the backbone remains stable, *T*_max_ is unchanged. However, the higher or comparable *T*_endset_ suggests that the overall degradation process extends to a higher temperature. Therefore, TRC modification broadens the degradation step rather than simply improving the initial degradation temperature.

The most beneficial effect appears more clearly in the degradation rate (*R*_max_), not in the initial degradation temperature. *R*_max_ decreased from 3.13 to 2.40%/°C for the PE-1 system, corresponding to about a 23% reduction in maximum degradation rate. Similarly, *R*_max_ decreased from 3.16 to 2.35%/°C for the PE-2 system, corresponding to an approximately 26% reduction. This suggests that the TRC systems degrade more gradually during the main decomposition stage. This slower degradation may be associated with restricted chain mobility and interchain coupling, which can delay the rapid volatilization of degradation fragments. The DTG-derived slope from *T*_onset_ to *T*_max_ provides additional support for this interpretation. This parameter describes how quickly the degradation rate increases after the onset of degradation until it reaches the maximum DTG peak. A higher magnitude value means that the material enters rapid degradation more abruptly, while a lower value indicates a more gradual acceleration of mass loss. For the neat PE samples, the DTG slope values were relatively high (i.e., 0.08%/°C^2^ for PE-1 and 0.10%/°C^2^ for PE-2). After TRC modification, these values decreased to 0.06%/°C^2^ for TRC-PE1 and 0.05%/°C^2^ for TRC-PE2. This reduction suggests that the modified TRC systems do not enter rapid degradation as abruptly as neat HDPE. Instead, the degradation process becomes more gradual, which is consistent with the lower *R*_max_ values observed for the TRC formulations. The TGA slope values support the same conclusion. For PE-1, the average degradation slope decreases in magnitude from −2.27%/°C for neat PE-1 to −1.81%/°C for TRC-PE1. For PE-2, it decreases from −2.41%/°C for neat PE-2 to −1.95%/°C for TRC-PE2. This confirms that the TRC systems undergo mass loss over a broader temperature interval and degrade less abruptly than neat HDPE. The *T*_max_ − *T*_onset_ value from DTG curve represents the temperature interval required for the sample to progress from the onset of degradation to the maximum degradation rate. A larger value means that degradation develops over a broader temperature window before reaching its maximum rate. For neat PE-1 and PE-2, the values are 37 and 38 °C, respectively. After TRC modification, these increase to 48 °C for TRC-PE1 and 50 °C for TRC-PE2. This is an important result because it shows that the TRC systems require a wider temperature interval to reach their maximum degradation rate. In other words, the degradation process is delayed or broadened after reactive modification.

Carbon black showed a formulation-dependent effect. In the PE-1 series, carbon black partially compensated for the decrease in early degradation temperature. TRC-PE1-CB0.5 showed a *T*_5%_ of 410 °C, which is higher than that of TRC-PE1 but still lower than that of neat PE-1. TRC-PE1-CB1 showed *T*_max_ comparable to that of neat PE-1 and a lower degradation rate than that of neat PE-1, suggesting that carbon black helped moderate the degradation process but did not fully restore early thermal stability. In the PE-2 series, the strongest beneficial response was observed at 0.5 wt% carbon black. TRC-PE2-CB0.5 exhibited the best thermal profile among the PE-2-based TRC systems, with a *T*_5%_ of 422 °C, *T*_10%_ of 439 °C, *T*_50%_ of 467 °C, and *T*_max_ of 473 °C. These values are slightly higher than those of neat PE-2, indicating that 0.5 wt% carbon black provides an effective stabilizing or barrier effect in this formulation. However, increasing carbon black to 1 wt% did not further improve the early degradation temperature. TRC-PE2-CB1 showed lower *T*_5%_ and *T*_10%_ temperatures, despite having the lowest *R*_max_. This suggests that higher CB loading may introduce dispersion-related heterogeneity or localized thermal effects.

Overall, the TGA and DTG results demonstrate that TRC-PE formulations retain the high-temperature degradation resistance of HDPE while exhibiting a slower and broader degradation process. Although the early degradation temperature decreases in some TRC systems, all formulations remain thermally stable far above the processing and printing temperatures. These results support the thermal suitability of the developed TRC-PE materials for filament extrusion and FDM 3D printing.

### 3.5. Tensile Testing

**Figure 7:**
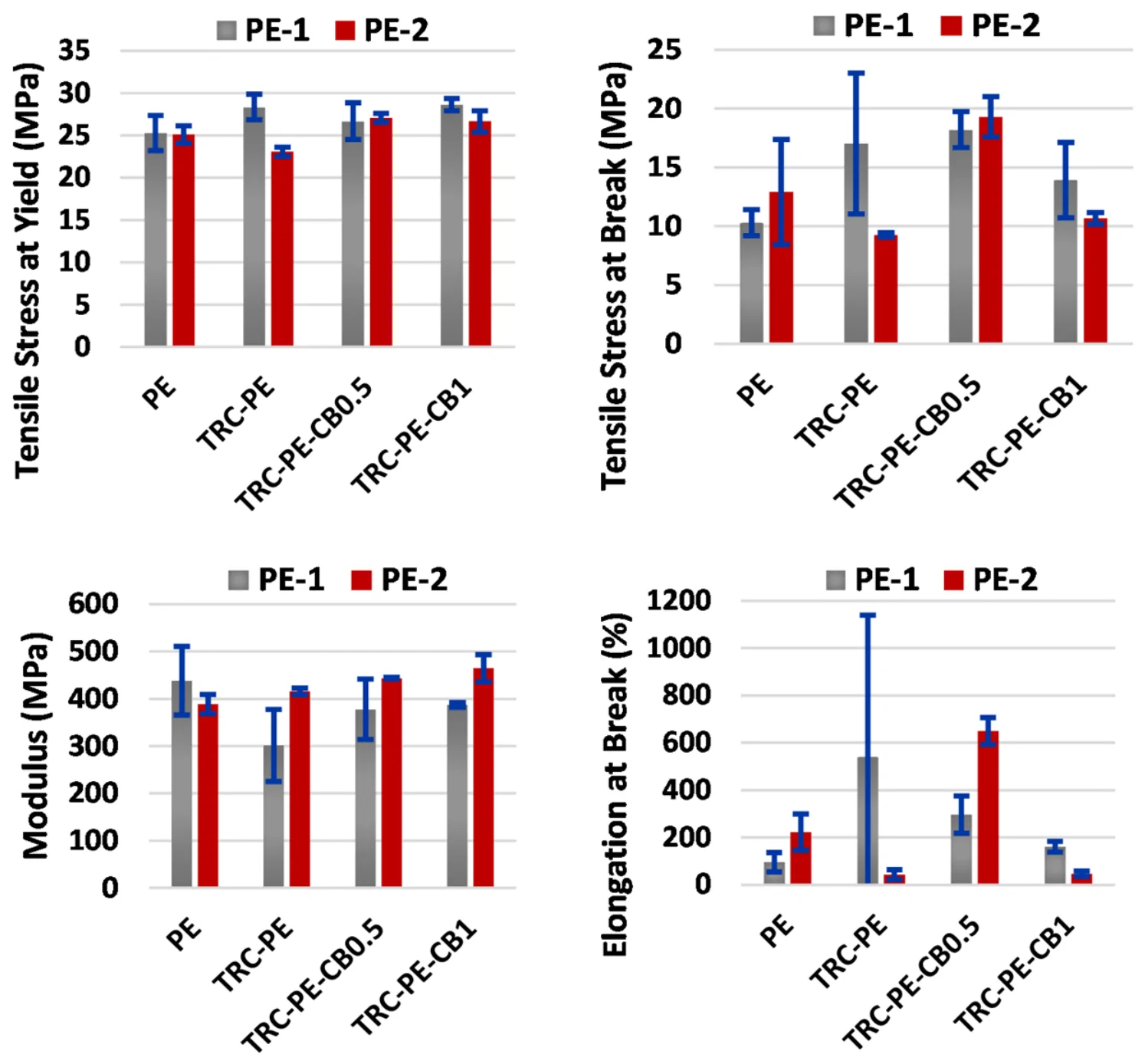
Tensile properties of 3D-printed PE-1- and PE-2-based TRC formulations.

**Figure 8:**
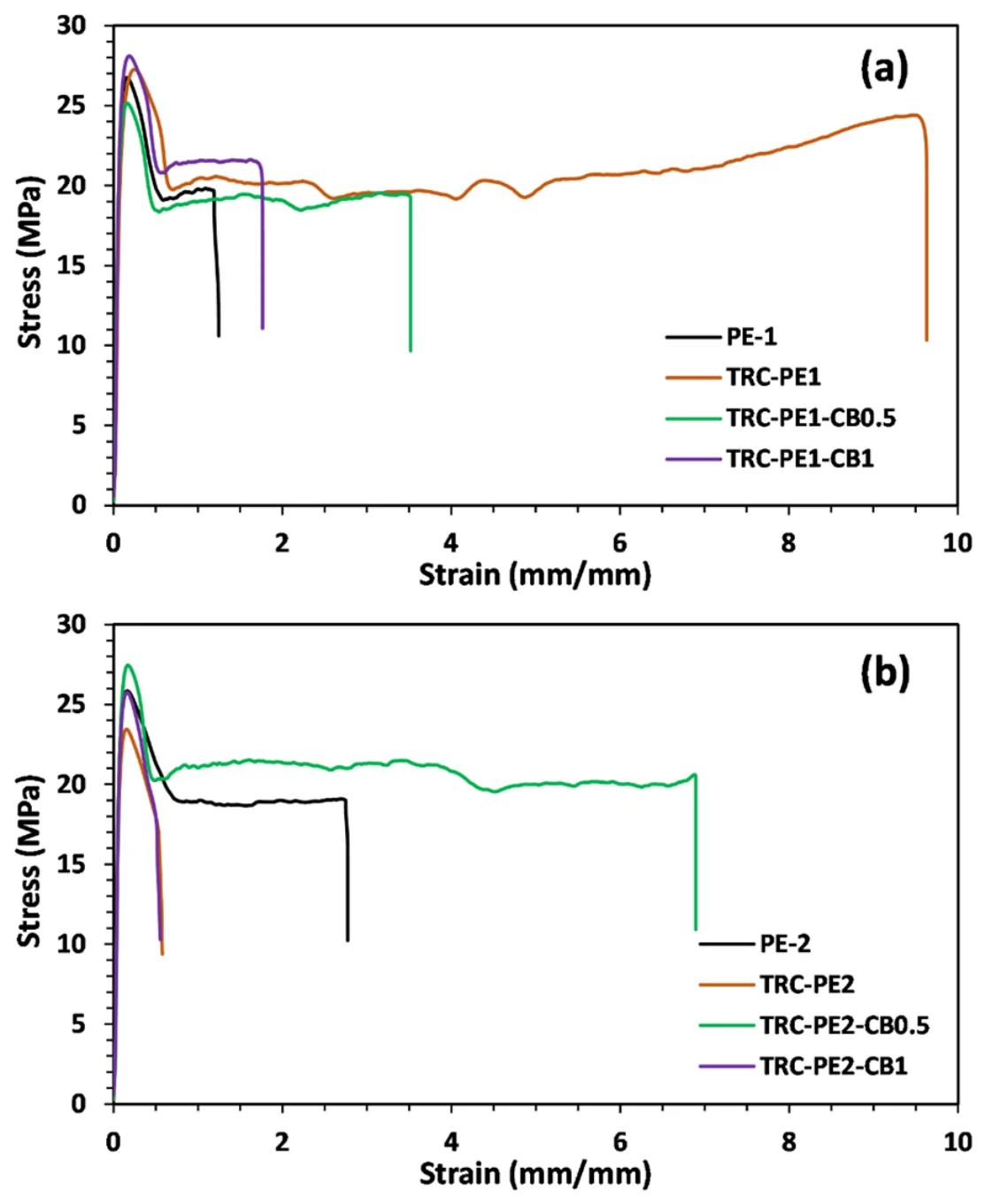
Stress–strain curves of 3D-printed (**a**) PE-1 and TRC-PE1 formulations, and (**b**) PE-2 and TRC-PE2 formulations.

The tensile properties of the 3D-printed neat PE and TRC-PE samples were evaluated to determine the effect of reactive modification and carbon black incorporation on the mechanical performance of the printed structures. The tensile characteristics of 3D-printed PE and TRC-PE systems are shown in Figure 7 and Figure 8. In the PE-1 series, neat PE-1 exhibited a tensile stress at yield of 25.29 ± 2.07 MPa, tensile stress at break of 10.31 ± 1.09 MPa, modulus of 438 ± 72 MPa, and elongation at break of 95% ± 41. After TRC modification, TRC-PE1 showed a clear increase in tensile stress at yield, tensile stress at break, along with the elongation at break compared with neat PE-1. The tensile stress at yield, tensile stress at break, and lastly the elongation at break increased by ~12%, ~65%, and ~466%, respectively, compared with neat PE-1. This strengthening is consistent with the formation of ester-containing interchain-coupled structures that may act as molecular tie points, increasing resistance to plastic deformation. However, the modulus decreased by ~31%, indicating that the reactive modification may partially disrupt crystalline packing than the increased chain connectivity contributes to elastic load transfer. This suggests that the TRC modification increased the ductility and toughness but reduced stiffness. Therefore, this improved ductility can help reduce brittle failure, crack propagation, and interlayer fracture for 3D-printed TRC-PE. The reactive modification increased the resistance to yielding without the need for particulate reinforcement. The incorporation of carbon black (CB) further influenced the mechanical response of the PE-1-based TRC systems, and the addition of 0.5 wt% carbon black to TRC-PE1 imparted the best balance in the PE-1 series. The composite TRC-PE not only improved the tensile stress at yield, tensile stress at break, as well as the elongation at break, but also partially restored the compromised modulus while simultaneously improving strength and ductility, indicating an effective reinforcing contribution from carbon black. The elongation at break is particularly sensitive to defects generated during processing and to the ability of the printed objects to undergo stable plastic deformation. Although TRC-PE1 showed a remarkable approximately 466% increase in the elongation at break, this was accompanied by an enormous standard deviation. The large scatter indicates that the material is capable of very high ductility, yet small processing variability led to premature failure in some specimens. Adding CB to TRC-PE1 reduces the average elongation while markedly improving the reproducibility (i.e., standard deviation). TRC-PE1-CB0.5 showed the highest tensile stress at break, increasing from 10.31 ± 1.09 MPa for PE-1 to 18.21 ± 1.52 MPa. It also maintained a substantially higher elongation at break of 296%, which is more than three times (i.e., a 210% increase) that of neat PE-1. This indicates that 0.5 wt% carbon black provided a favorable balance between reinforcement and ductility, which resulted in an effective balance between reinforcement and ductility without excessively restricting chain mobility. In contrast, increasing the CB content to 1 wt% further enhanced yield stress but lowered stress at break and elongation as compared with CB0.5 (but still much better than that of neat PE-1), suggesting that excessive CB may restrict ductile deformation or introduce stress-concentration sites. The addition of carbon black to TRC-PE1 also reduced the standard deviation in elongation at break, unlike that observed in TRC-PE1.

A different tensile behavior was observed for the TRC-PE2 in PE-2 series. TRC-PE2 without carbon black showed lower tensile stress at yield, lower tensile stress at break, and a substantial reduction in elongation at break compared with neat PE-2. This embrittlement could be caused by several possible factors, including differences in molecular architecture, variations in the extent of interchain coupling during reactive extrusion resulting in uneven network formation, or localized degradation reactions that shorten chains and suppress the large-scale plastic drawing required for high elongation. This behavior indicates that TRC modification alone was not sufficient to improve the mechanical performance of the PE-2 system. The reduced ductility may also be associated with differences in the molecular architecture, melt flow behavior, crystallization tendency or degree of reactive modification. However, these possibilities cannot be distinguished conclusively from the present characterization. It may be possible that a different weight loading of the additive could yield better mechanical performance. Therefore, the introduction of reactive modification alone may be accompanied by changes in crystalline perfection or by chain scission during reactive extrusion, which could have affected the glassy-amorphous contribution to yield. These proposed explanations remain tentative, as direct evidence was not obtained in this study, which is beyond the scope of the present work.

Similar improved tensile properties are observed for TRC-PE2-CB0.5, which showed simultaneous improvement in all major tensile parameters. For example, its tensile stress at yield increased by 7.8%, tensile stress at break increased by 49.5%, its Young’s modulus increased by 14.0%, and elongation at break increased by 191.5% compared with neat PE-2. This is a notable outcome because a simultaneous improvement in stiffness, strength, and elongation is rarely observed in filled polyethylene systems, where enhancements in modulus and yield stress typically come at the expense of ductility [42]. This suggests that 0.5 wt% loading of carbon black may also have influenced the processing, improving the dispersion of the reactive modification chemistry or scavenging radicals to suppress degradation, thereby resulting in a more uniform distribution of ester-containing interchain-coupled structures throughout the material. Direct experimental observation is the significant mechanical improvement upon CB addition; the specific mechanistic roles proposed above are hypotheses consistent with our tensile data and the established radical-scavenging and nucleating behavior of carbon black reported in the literature. Therefore, 0.5 wt% CB provided an optimal reinforcing effect while also improving the overall quality of printed structures as evidenced by smoother surface morphology and fewer visible printing defects based on the macroscopic visual appearance of the printed specimens, possibly through improved thermal conductivity because of CB addition, leading to more uniform heat distribution, and reduced shrinkage-induced defects during printing. The CB filler then compensates by reinforcing the ester-containing interchain-coupled polyethylene structure and possibly nucleating a higher density of smaller crystallites that raise the yield point. At higher CB loading in the PE-2 system, stiffness continued to increase, but ductility and stress at break decreased sharply. This indicates that the higher CB loading may over-reinforce the system, restrict chain mobility, or promote local defects/agglomeration. Overall, the tensile results demonstrated that the optimized TRC-PE/carbon black formulations significantly improved the mechanical performance of 3D-printed HDPE. The most promising formulation was TRC-PE2-CB0.5, which showed simultaneous enhancement in tensile stress at yield, stress at break, modulus, and elongation at break. In the PE-1 series, TRC-PE1-CB0.5 also provided the highest stress at break and substantially improved elongation compared with neat PE-1. In the composite TRC-PE-CB, CB may act as a processing stabilizer by adsorbing radical species and excess catalyst, moderating the extent of crosslinking and preventing local over-crosslinked domains. CB dispersion may also impart a nucleating effect where a fine CB dispersion can increase nucleation density, resulting in smaller, more uniform spherulites that enhance drawability. CB may also act as network reinforcement, where CB aggregation could lead to stress localization, crack initiation, and premature failure, explaining the return to brittleness. Overall, the results support the effectiveness of the reactive modification strategy in enhancing the mechanical performance as well as the printing characteristics of modified HDPE-based materials, particularly when combined with an optimized low loading of CB. The thermal analysis findings also align closely with these tensile results and together reinforce the structure–property relationships in the modified HDPE formulations.

Taken together, the experimental findings establish a coherent structure–property–performance relationship for the modified HDPE system. The ^1^H NMR and FTIR data provide evidence for successful maleic anhydride grafting and the formation of ester-containing interchain-coupled polyethylene structures, which are consistent with the DSC observation of a preserved yet slightly constrained crystalline phase while retaining melt-processability, and reduced visible shrinkage observed in the modified formulations. The TGA results further revealed a wide safety margin for processing together with a broadened degradation profile and diminished maximum mass-loss rate, indicative of possible restricted segmental mobility resulting from ester-linked interchain structures. This same restriction is consistent with the observed enhanced load distribution and interfacial bonding, which likely contributed to the simultaneous gains in yield stress, stress at break, and elongation seen in the optimized TRC-PE2-CB0.5 composition. Notably, the TGA results for this formulation, specifically its recovery of early degradation temperatures to levels matching or exceeding neat HDPE, parallel its superior tensile performance, suggesting that the addition of carbon black at 0.5 wt% not only reinforces the matrix but also protects the ester-linked structures against the chain-scission reactions responsible for the brittleness of the unfilled TRC-PE2.

**Figure 9:**
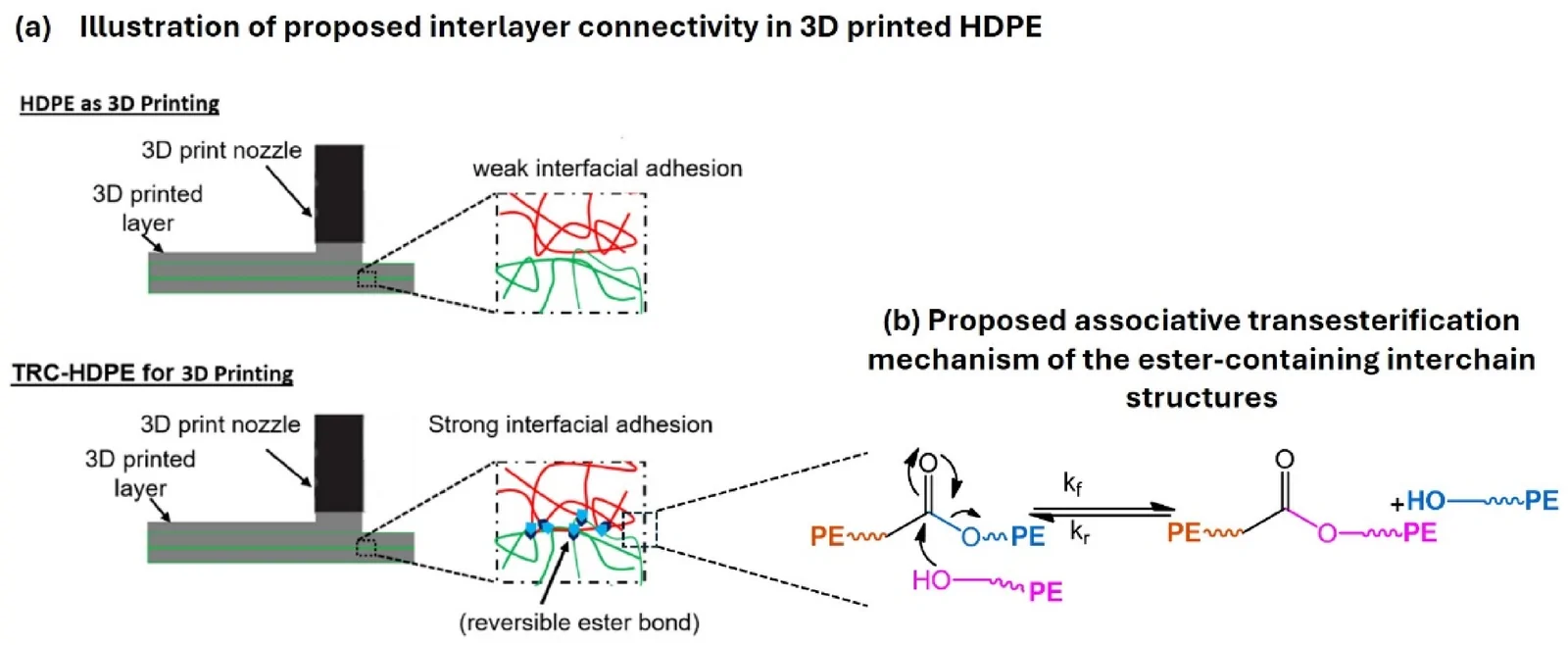
Proposed mechanism for the improved printability of the modified HDPE formulations. (**a**) Schematic illustration of the hypothesized enhancement in interlayer bonding resulting from ester-containing interchain structures. Green and red lines represent polyethylene chains originating from the two adjacent layers during 3D printing, while blue dots indicate interlayer connectivity. (**b**) Proposed associative ester-exchange pathway that could occur at elevated processing temperatures. The mechanism is presented as a conceptual framework for interpreting the observed improvements in filament consistency and mechanical performance.

Based on these experimental observations and well-established ester-exchange chemistry catalyzed by zinc salt [43,44], a proposed transesterification-based dynamic exchange mechanism for the ester-linked interchain structures is illustrated in Figure 9. to rationalize these collective improvements in printability and mechanical performance. The pre-designed additive used to modify HDPE contains zinc acetate, which serves as a transesterification catalyst for ester-linked structures, and could therefore provide the chemical basis for potential dynamic exchange capability to the network, while 4-hydroxy-TEMPO acts as a radical scavenger to suppress undesired side reactions such as chain scission or uncontrolled crosslinking. The bis-stearamide acts as a co-agent to suppress irreversible crosslinking in polyethylene. Based on well-established zinc-catalyzed transesterification chemistry, the dynamic covalent bond exchange could occur via transesterification between the ester linkages and the residual hydroxyl or carboxyl groups, catalyzed by zinc acetate. However, the total concentration of residual hydroxyl groups in the final modified HDPE is expected to be low and dispersed within a predominantly non-polar polyethylene matrix to clearly resolved in the solid-state FTIR spectra. At elevated processing temperatures, the molecular mobility and molecular contact substantially increase; the ester-containing interchain structures may undergo associative transesterification reactions, permitting temporary rearrangement of the molecular architecture before becoming increasingly restricted upon cooling. Such dynamic rearrangement could facilitate stress relaxation during extrusion and printing while contributing to improved printing quality after deposition. Likewise, the proposed ester-containing interchain structures may promote improved connectivity between deposited filaments than is achieved via the weak van der Waals interactions typical of neat HDPE, thus providing one possible explanation for the enhanced tensile performance observed in the optimized formulations. While direct verification of dynamic bond exchange and its influence on interlayer bonding is beyond the scope of this study and would require dedicated rheological, stress-relaxation, swelling, or interlayer adhesion studies, the observed combination of improved filament consistency, enhanced print quality, and simultaneously enhanced strength and ductility is fully consistent with such a dynamic network.

These results demonstrate that the ester coupling approach, likely operating through the proposed dynamic exchange, effectively improves the printability and quality of HDPE and provides a promising strategy to address the two fundamental barriers that have excluded polyethylene from mainstream fused deposition modeling: severe warpage and weak interlayer fusion. By transforming unprintable commodity HDPE into a consistent, high-performance filament through a single-step, solvent-free process, this work opens a practical pathway for valorizing post-consumer polyolefin waste in the rapidly growing additive manufacturing sector. The ability to simultaneously enhance stiffness, strength, and ductility while retaining full melt reprocessability positions this modified HDPE as a compelling candidate for sustainable 3D-printing applications.

## 4. Conclusions

Based on the experimental findings, ester-containing interchain coupling is an effective strategy for rendering HDPE suitable for 3D printing while preserving its melt-processability. Single-step reactive extrusion of HDPE with maleic anhydride and 1,10-decanediol, catalyzed by zinc acetate, generated ester-linked interchain structures, as confirmed by ^1^H NMR and FTIR analyses. Unlike neat HDPE, which yielded irregular filaments and poor prints, the modified formulations produced filaments of consistent diameter and printed successfully with reduced visible warpage. DSC confirmed the retention of well-defined polyethylene melting transitions (131–136 °C) with a modest decrease in melting enthalpy, indicating that interchain coupling restricts chain packing without destroying the crystalline phase. All formulations remained thermally stable well above processing temperatures. The optimized ester-coupled/carbon black formulations improved tensile stress at break by approximately 50–75% and elongation at break by approximately 200% relative to neat HDPE. Collectively, these findings demonstrate a simple, single-step, and commercially viable route to additive-manufacturing feedstock that avoids specialty crosslinkers and hazardous reagents—thus offering a pathway for valorizing pigmented HDPE waste, which is otherwise unsuitable for packaging reuse, from landfill. Future work should verify the proposed transesterification mechanism through stress-relaxation rheology and extend the approach to post-consumer HDPE.

## Figures and Tables

**Table 1:** Sample code designations.

Sample Code	Nature	Composition (wt% Loading in HDPE)	
		Additive	Carbon Black
PE-1	Neat commercial HDPE 1	-	-
TRC-PE1	Thermo-reactively modified HDPE	5	-
TRC-PE1-CB0.5	Thermo-reactively modified HDPE + CB 0.5%	5	0.5
TRC-PE1-CB1	Thermo-reactively modified HDPE + CB 1%	5	1
PE-2	Neat commercial HDPE 2	-	-
TRC-PE2	Thermo-reactively modified HDPE	5	-
TRC-PE2-CB0.5	Thermo-reactively modified HDPE + CB 0.5%	5	0.5
TRC-PE2-CB1	Thermo-reactively modified HDPE + CB 1%	5	1

CB stands for carbon black, and TRC stands for Thermo-Reactive Coupling (heat-driven reactive extrusion, interchain coupling).

**Table 2:** Thermal profile of the neat PE and TRC-PE samples obtained from DSC curves.

Sample Code	Melting Transition (Endothermic Peak) and Melting Profile			
	*T*_onset_ (°C)	*T*_m_ (°C)	*T*_endset_ (°C)	Δ*H*_m_ (J/g)
PE-1	124.1	133.7	137.7	208.1
TRC-PE1	124.0	135.1	137.9	196.4
TRC-PE1-CB0.5	122.7	131.3	135.9	196.9
TRC-PE1-CB1	122.5	131.6	136.4	203.1
PE-2	125.6	136.5	139.1	193.9
TRC-PE2	125.9	135.7	140.2	184.5
TRC-PE2-CB0.5	123.4	132.5	135.7	214.1
TRC-PE2-CB1	124.1	133.7	137.7	208.1

**Table 3:** Thermal profile of the neat PE and TRC-PE samples from TGA curves.

Sample Code	DTG Curves						TGA Curves					
	*T*_onset_ (°C)	*T*_max_ (°C)	*R*_max_ (%/°C)	Slope (After *T*_onset_ to *T*_max_) (%/°C^2^)	*T*_max_ − *T*_onset_ (°C)	*T*_5%_ (°C)	*T*_10%_ (°C)	*T*_50%_ (°C)	*T*_onset_ (°C)	*T*_endset_ (°C)	Slope (*T*_onset_ to *T*_endset_) (%/°C) ^a^	*T*_endset_ − *T*_onset_ (°C)
PE-1	435	472	3.13	0.08	37	418	431	463	447	480	−2.27	33
TRC-PE1	423	471	2.40	0.06	48	400	422	462	438	484	−1.81	46
TRC-PE1-CB0.5	432	470	2.57	0.06	38	410	436	462	444	482	−2.15	38
TRC-PE1-CB1	432	472	2.58	0.06	40	405	431	464	445	484	−2.10	39
PE-2	434	472	3.16	0.10	38	420	433	464	448	481	−2.41	33
TRC-PE2	421	471	2.35	0.05	50	404	431	463	443	483	−1.95	40
TRC-PE2-CB0.5	427	473	2.89	0.08	46	422	439	467	448	484	−2.29	36
TRC-PE2-CB1	424	471	2.33	0.05	47	399	423	462	438	483	−1.80	45

^a^ The negative sign indicates mass loss, and lower absolute magnitude means less abrupt degradation. Abbreviations and parameters are defined in the Materials and Methods and Abbreviations sections.

## Data Availability

The original contributions presented in this study are included in the article. Further inquiries can be directed to the corresponding author.

## Abbreviations

The following abbreviations are used in this manuscript:

^1^H NMR	Proton nuclear magnetic resonance
CB	Carbon black
DSC	Differential scanning calorimetry
DTG	Derivative thermogravimetric analysis
FDM	Fused deposition modeling
FFF	Fused filament fabrication
FTIR	Fourier-transform infrared
HDPE	High-density polyethylene
PE	Polyethylene
rHDPE	Recycled high-density polyethylene
*R*_max_	Maximum rate of degradation
TGA	Thermogravimetric analysis
*T*_m_	Melting temperature
*T*_max_	Temperature at the maximum degradation rate
*T*_m-endset_	Endset melting temperature
*T*_m-onset_	Onset melting temperature
*T*_endset_	Endset degradation temperature
*T*_onset_	Onset degradation temperature
TRC	Thermo-reactively coupled
Δ*H*_m_	Enthalpy change of melting
